# A Scooping-Binding Robotic Gripper for Handling Various Food Products

**DOI:** 10.3389/frobt.2021.640805

**Published:** 2021-03-26

**Authors:** Zhongkui Wang, Haruki Furuta, Shinichi Hirai, Sadao Kawamura

**Affiliations:** ^1^ Research Organization of Science and Technology, Ritsumeikan University, Kusatsu, Japan; ^2^ Graduate School of Science and Engineering, Ritsumeikan University, Kusatsu, Japan; ^3^ Department of Robotics, Ritsumeikan University, Kusatsu, Japan; ^4^ Chitose Robotics Inc., Tokyo, Japan

**Keywords:** soft gripper, scooping, binding, food handling, grasping, slippery

## Abstract

Food products are usually difficult to handle for robots because of their large variations in shape, size, softness, and surface conditions. It is ideal to use one robotic gripper to handle as many food products as possible. In this study, a scooping-binding robotic gripper is proposed to achieve this goal. The gripper was constructed using a pneumatic parallel actuator and two identical scooping-binding mechanisms. The mechanism consists of a thin scooping plate and multiple rubber strings for binding. When grasping an object, the mechanisms actively makes contact with the environment for scooping, and the object weight is mainly supported by the scooping plate. The binding strings are responsible for stabilizing the grasping by wrapping around the object. Therefore, the gripper can perform high-speed pick-and-place operations. Contact analysis was conducted using a simple beam model and a finite element model that were experimentally validated. Tension property of the binding string was characterized and an analytical model was established to predict binding force based on object geometry and binding displacement. Finally, handling tests on 20 food items, including products with thin profiles and slippery surfaces, were performed. The scooping-binding gripper succeeded in handling all items with a takt time of approximately 4 s. The gripper showed potential for actual applications in the food industry.

## 1 Introduction

In recent years, there has been an increasing demand for automation in the food industry, agriculture, forestry, and fisheries due to labor shortages. In food factories, suction pads are widely used for handling packaged food products in automated production lines. However, there are many food products, such as raw and fried foods, vegetables, and fishes, that cannot be handled by suction pads. For such food products, human laborers usually perform handling tasks. The lack of effective robotic end-effectors is one of the main reasons why automation in the food industry is not as developed as in the automobile and electronics industries. To handle food products using robots, robotic end-effectors must adapt to objects with large variations in shape, size, softness, and surface conditions (Caldwell et al., 2009). This poses challenges to current industrial robotic hands or grippers.

To address the above-mentioned difficulties, novel robotic grippers were proposed and applied to handle various types of food products. A soft gripper exploiting the effects of magnetorheological fluid was proposed to adapt to the differences in shape and softness of vegetables and fruits, such as apples, carrots, strawberries, and broccolis (Pettersson et al., 2010). A hygienically designed force gripper was also developed by the same authors for handling variable and easily damaged food products (Pettersson et al., 2011). This gripper consists of two parallel-configured thin fingers, and magnetic coupling was used to transfer the linear motion to encapsulate the actuator mechanism completely. A robotic end-effector was developed according to the Bernoulli principle for handling sliced vegetables; it was tested on sliced tomato and cucumber for assembling sandwich (Davis et al., 2008). A robotic gripper combining the Bernoulli principle and multi-fingered grasping was also proposed in (Sam and Nefti, 2010) for handling variable size, shape, and weight of unpacked food products. Endo and Otomo developed a two-degree-of-freedom multi-fingered gripper for dishing up noodles and simmered foods while considering an appetizing presentation (Endo and Otomo, 2016). Li et al. proposed an origami “magic-ball” soft gripper that could grasp objects much heavier than the gripper itself (Li et al., 2019). The gripper was tested by grasping 12 food items. A lightweight kirigami gripper was also developed by Ma et al. for food grasping (Ma et al., 2020). The gripper weighs 4 g and was able to handle an object of 25 g. Gafer et al. proposed a cable-driven quad-spatula gripper and experimentally demonstrated its capability for grasping food ingredients (Gafer et al., 2020). A robotic hand with fluid fingertips and a fluid pressure monitoring-based grasping strategy were introduced in (Nishimura et al., 2019) for grasping fragile objects, such as a tofu, a sushi, and a potato chip. A soft robotic gripper with enhanced object adaptation and grasping reliability was also proposed in (Zhou et al., 2017), and it was experimentally tested by grasping various objects including fruits and foot materials. Wang et al. proposed a series of grippers for handling food and agricultural products, such as a 3D printable soft gripper and a pre-stressed gripper for packaging lunch boxes (Wang et al., 2016; Wang et al., 2017), a wrapping gripper for handling granular foods (Kuriyama et al., 2019), a circular shell gripper for grasping and twisting (Wang et al., 2020a), a soft gripper equipped with suction cups to realize both grasping and suction modes (Wang et al., 2020b), a needle gripper for grasping chopped food material, such as salads, and eliminating defective products by piercing (Makiyama et al., 2020), and a parallel shell gripper for packaging multiple cucumbers simultaneously (Kanegae et al., 2020).

Aside from handling food products, soft grippers were also proposed for handling various daily objects. A universal gripper based on jamming principle was proposed by Brown et al. It can handle a wide range of different objects, such as a shock absorber coil, screw driver, bottle caps, plastic tubing, and so on (Brown et al., 2010). The jamming principle was also utilized to develop soft grippers with variable stiffness (Li et al., 2017; Zhu et al., 2019). Bioinspired soft grippers were developed to conform to the object shape with passive grip by mimicking the Manduca sexta (Crook et al., 2017) and generate large grasping force by mimicking the winding behaviors of phythons and vines (Li et al., 2020). Anthropomorphic soft gripper and hand were proposed to achieve adaptable, effective, and dexterous grasping (Manti et al., 2015; Deimel and Brock, 2016). Soft grippers integrated novel adhesive interfaces with soft structures were investigated to enhance grasping capability (Song et al., 2017; Hao et al., 2020). Gerez et al. developed a soft and a hybrid grippers based on the soft, retractable, pneumatically actuated, telescopic actuators for handlinig fragile, delicate, heavy, and irregular objects (Gerez et al., 2020). Moreover, embedded soft sensing was also investigated to provide feedback during contact between soft gripper and objects (Wang and Hirai, 2016; Zhao et al., 2016; Truby et al., 2018).

In addition to research studies, some soft grippers were commercialized to improve automation in the food industry and agriculture, such as the mGrip gripper from (Soft Robotics Inc, 2020), the adaptive-shape soft gripper from (Festo, 2020), the soft gripper from (OnRobot A/S , 2020), the SOFTmatics gripper from (Nitta Co. Ltd., 2020), the modular-designed soft gripper from (SoftGripping, 2020), and the flexible gripper for harvesting tomato from (Root AI, 2020). These above-mentioned grippers demonstrate the potential for handling various types of food, agricultural products, and daily objects. However, these promising grippers still have difficulties in handling food products with thin profiles, and the handling of very slippery products, such as fish or other seafood, has not been addressed frequently so far. In addition, conventional robotic hands and grippers tend to avoid the contact with the external environment while grasping, and they usually lift objects by using friction force mainly. This is one of the possible reasons why food products with thin profiles and slippery surfaces are difficult for conventional grippers to handle.

In this study, we propose a scooping-binding gripper to handle various food products, including products with thin profiles and slippery surfaces. This is an extension of our previous binding hand (Iwamasa and Hirai, 2015; Okada et al., 2019), in which a single flexible string was used to grasp target object. In the scooping-binding gripper, we adopted a parallel gripper structure and increased the number of binding strings. To grasp objects with thin profiles and slippery surfaces, we integrated two thin and slim plates at the gripper bottom. Upon grasping, the thin plates can be inserted under the target object to provide a supporting force from the target bottom. Simultaneously, the flexible strings wrap around the target object to secure a stable grasp during high-speed motions. The main contributions of this study are 1) proposing a novel robotic gripper for handling various food products, and 2) experimental validation of the gripper for handling food products with thin profiles and slippery surfaces.

The remainder of this paper is organized as follows. The gripper concept, design, and fabrication are introduced in Section 2, followed by the contact analysis in Section 3. The caracterization of the flexible string and the analysis of the binding force were investigated in Section 4. Handling tests on various food products are presented in Section 5. Section 6 concludes the paper and suggests future work.

## 2 Scooping-Binding Gripper

### 2.1 Concept

**FIGURE 1:**
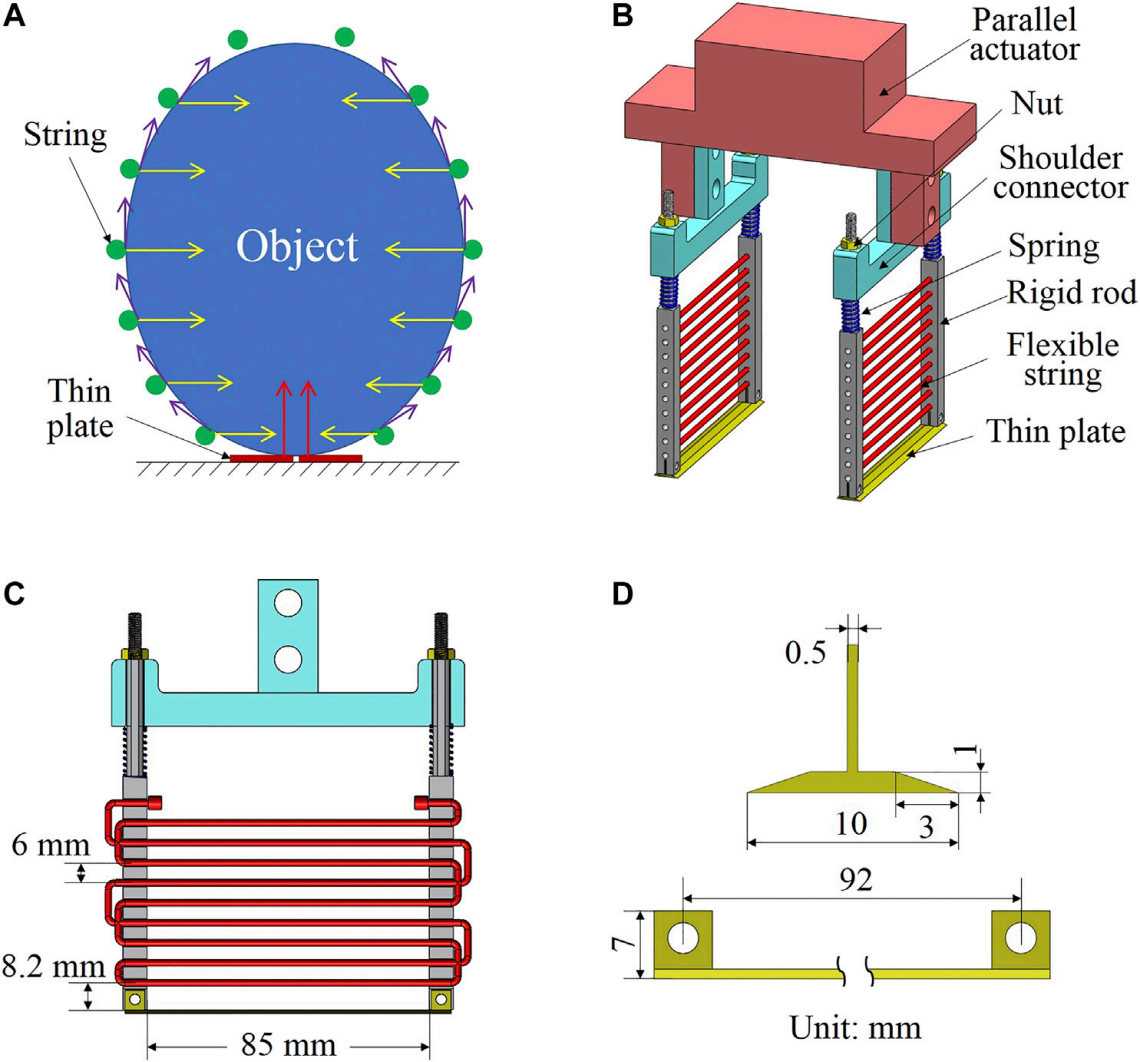
Proposed scooping-binding gripper. **(A)** shows the concept, **(B)** depicts the CAD design of the gripper, **(C)** presents a section view of the gripper showing the dimensions and the string winding, and **(D)** depicts the design and dimensions of the thin plate.

The idea of binding gripper was reported in our previous work Iwamasa and Hirai (2015); Okada et al. (2019). It uses a single flexible string to form a closed loop for grasping and lifting object. It can adapt to objects with variable shapes and sizes because of the string flexibility. As long as the object is located in the closed loop of the gripper, it can be grasped by winding the string while closing the rigid rods using only one motor. However, binding grippers have difficulties in grasping thin, heavy, and slippery objects. Therefore, we propose a scooping-binding gripper to handle such objects. The concept is shown in Figure 1A. There are two main differences between the scooping-binding gripper and the original binding gripper. The first one is that we adopted a parallel configuration and used multiple strings to provide larger grasping force (yellow arrows) and friction force (purple arrows). Multiple strings can generate a 3D wrapping for better stabilization of the grasping. The second difference is that we placed two thin plates (red thick lines) at the gripper bottom and they can be inserted under the object bottom to provide support force (red arrows). When grasping thin, heavy, and slippery objects, the thin plates play a central role in supporting the object weight, and the strings are responsible for stabilizing the grasped object to avoid dropping during high-speed pick-and-place motions. One important feature of the scooping-binding gripper is that it requires active contact with a table or conveyor where the target object is placed on.

### 2.2 Gripper Design

Based on the above concept, we designed the scooping-binding gripper (see Figure 1B). The gripper was constructed using a pneumatic parallel actuator and two identical scooping-binding mechanisms. This mechanism consists of a shoulder connector, two rigid rods, two springs, two nuts, a thin plate, and multiple strings between the two rigid rods. Given that the mechanism has to make contact with the environment, two springs are employed to maintain constant contact and also absorb the impact when contact happens. The rigid rod has multiple holes to allow the string to pass through. The nut is used to connect the rod to the shoulder connector and adjust the preload of the spring. A slot is designed at the bottom end of the rod to assemble the thin plate. To fix the string to the rods, we adopted a winding approach and used one single string to wind through both rods (see Figure 1C). The interval between the neighboring strings is 6 mm. The design of the thin plate is shown in Figure 1D. It has a width of 10 mm and a thickness of 1 mm with a slope length of 3 mm.

### 2.3 Fabrication and Assembly

**FIGURE 2:**
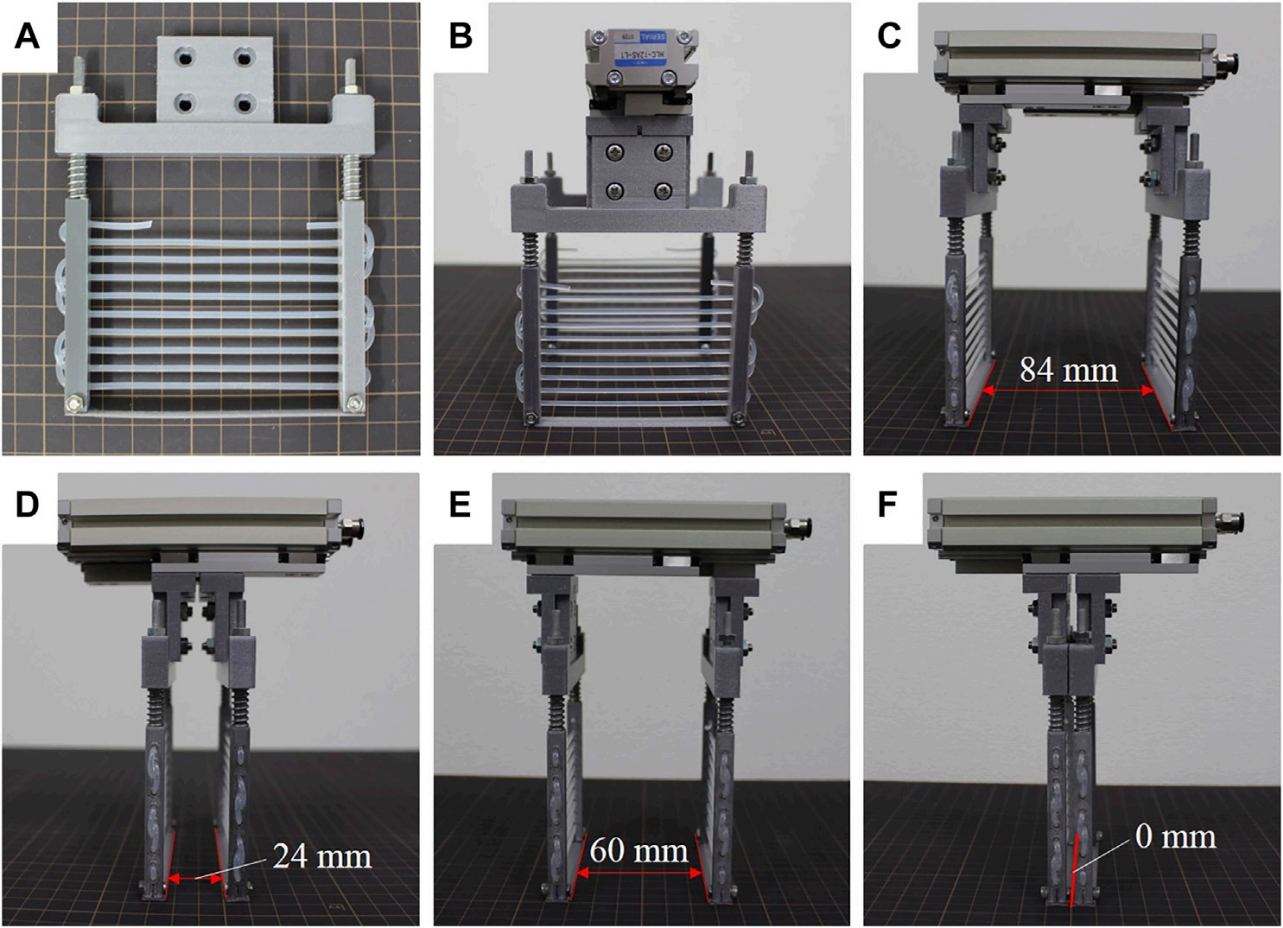
Fabricated scooping-binding gripper. **(A)** shows the scooping-binding mechanism, **(B)** depicts the assembled gripper, **(C)** and **(D)** show the open and close states in wide configuration, and **(E)** and **(F)** show the open and close states in narrow configuration.

The shoulder connector, rigid rods, and thin plate were 3D printed using a 3D printer (Prusa I3 MK3, Prusa Research, Prague) and PLA (polylactic acid) material. They were then assembled together with the springs, nuts, and string to construct one scooping-binding mechanism (see Figure 2A). The spring has a free length of 19 mm, an external diameter of 7.5 mm, and a spring constant of 2.55 N/mm. A silicone rubber round string (3-2316-02, AS ONE, Osaka) was chosen as the thin string and winded through the holes on the rigid rods. The rubber string has a diameter of 3 mm and a hardness of Shore A55. A commercially available pneumatic parallel actuator (HLC-12AS-L1, Kondoh Mfg.Co.,Ltd., Nagoya) was used to realize the parallel open and close motions; it has a stroke of 60 mm. The scooping-binding mechanisms were connected to the parallel actuator using a base flange to complete the assembly of the scooping-binding gripper (see Figure 2B). Depending on the fixture of the scooping-binding mechanisms, the gripper can have different widths upon opening and closing. For the wide configuration in which the scooping-binding mechanisms are connected to the external sides of the base flange, the gripper presents an opening width of 84 mm (see Figure 2C) closing width of 24 mm (see Figure 2D). By contrast, the gripper has a 60-mm (see Figure 2E) opening and 0-mm (see Figure 2F) closing widths for the narrow configuration in which the scooping-binding mechanisms are connected to the internal sides of the base flange. The gripper has a total weight of 536.8 g including the pneumatic parallel actuator, which weighs 440 g.

## 3 Contact Analysis

An important feature of the gripper is that it requires the scooping-binding mechanism to make contact actively and then sliding on the external environment during grasping. Upon sliding, the scooping-binding mechanism is subject to a bending moment that may deflect the rigid rods and therefore hinder the grasping motion. To this end, contact analysis was conducted based on both the simple beam theory and the finite element method.

### 3.1 Beam Model Analysis

**FIGURE 3:**
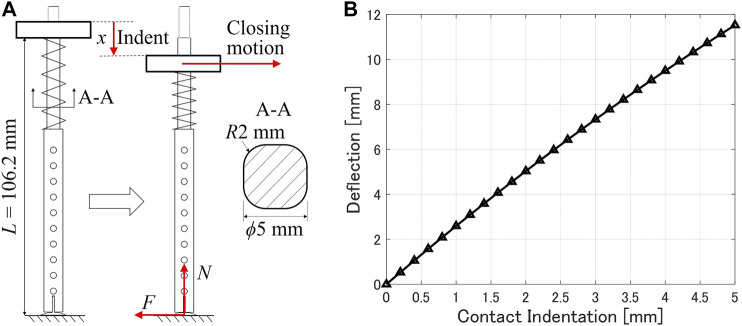
**(A)** Beam model of the scoop-binding mechanism to calculate the bending deflection. **(B)** Calculated bending deflection as a function of the contact indentation.

To study the bending behavior of the scooping-binding mechanism, we considered it a simple beam bending problem. As shown in Figure 3A, when the mechanism is vertically indented with a displacement of *x* (contact indentation), a contact force N=kx in the vertical direction is generated at the end of the rigid rod, where *k* is the spring constant. While closing the gripper, the shoulder connector generates a horizontal motion. Consequently, a friction force F=μN acts at the end of the rigid rod, where *µ* is the friction coefficient. This friction causes the bending deflecton of the rod. Based on the simple beam theory, the bending deflection *d* of the rod at an arbitrary indentation *x* can be calculated as

$$d=\frac{\mu k{\left(L-x\right)}^{3}}{3EI}x,$$ (1)

where *L* is the length of the rigid rod, *E* denotes the Young’s modulus of the rod material, and *I* is the moment of inertia, which depends on the cross-section of the rod. In our calculations, the rod material was PLA and the cross-section was a rounded square with its side length of 5 mm and round corner radius of 2 mm. The parameter values required for the calculations are listed in Table 1. A friction coefficient of 0.34 was measured between a thin plate made of PLA and a stainless surface using the inclined plane method. The calculated bending deflection as a function of the contact indentation *x*, is shown in Figure 3B. It suggests an approximately linear relationship. The deflection reached 11.53 mm at a contact indentation of 5 mm.

**TABLE 1:** Parameter values to calculate bending deflection.

*L* [mm]	*k* [N/mm]	*μ*	*E* [MPa]	*I* [mm^4^]
106.2	2.55	0.34	3500 Zhang et al. (2020)	37.125

### 3.2 Finite Element Analysis

**FIGURE 4:**
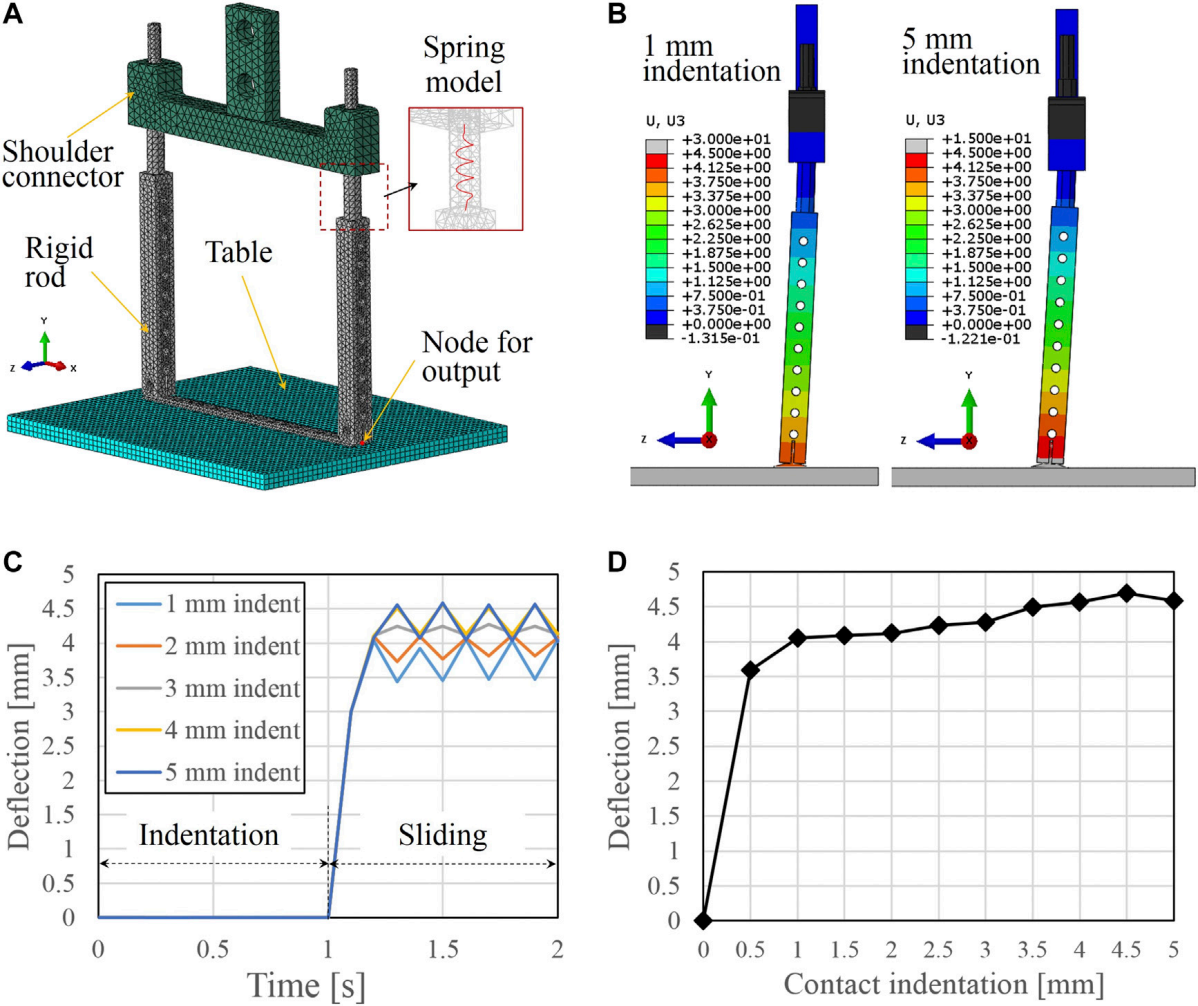
**(A)** Finite element model of the scoop-binding mechanism contacting a stainless table. **(B)** Examples of the simulated bending deflection of the rigid rod at indentations of 1 and 5 mm. **(C)** Simulation results of the bending deflection vs. time at different indentations from 1 mm through 5 mm. **(D)** Comparison of the simulated bending deflection at different contact indentations.

To reproduce the contact scenario better and simulate the actual structure of the scooping-binding mechanism, finite element analysis was conducted using Abaqus (SIMULIA, Dassault System, MA). The constructed finite element model is shown in Figure 4A. The same parameter values for *k*, *μ*, and *E* listed in Table 1 were used in this model. The external contact environment was modeled as a stainless table with a Young’s modulus of 190 GPa. The penalty method was used to model the contact interaction between the thin plate and the stainless table with a friction coefficient of 0.34. The friction coefficient between the shoulder connector and the rigid rods was set to 0.6 according to Perepelkina et al. (2017) because both parts were modeled as PLA material. The spring was modeled as “engineer feature of spring” in Abaqus with a spring constant of 2.55 N/mm; this spring connects the shoulder connector and the rigid rod in a similar manner to the gripper prototype. The model was meshed with 10-node quadratic tetrahedron elements. Initially, the thin plate and the stainless table were aligned at the same position in the Y-axis direction.

The simulations were conducted in two separate steps. In the first step, the stainless table was translated a predetermined vertical displacement along the Y-axis direction to indent the scooping-binding mechanism. Then, in the second step, the table was translated a horizontal distance of 30 mm along the Z-axis direction. The shoulder connector was fixed in space throughout the simulation. These two steps were used to simulate the actual scenario of grasping, but we translated the stainless table instead of the shoulder connector for convenience in the calculation of the bending deflection of the rigid rod. A node at the bottom center of the thin plate was set as an output node (see Figure 4A) to record the bending deflection of the rigid rod. The displacement component in the Z-axis direction of the output node was defined as the bending deflection.

Two examples of the simulated rigid rod deformation are shown in Figure 4B at indentation of 1 and 5 mm, respectively. We can observe the differences in the indentations and the bending deflections. Figure 4C compares the bending deflections of the output node as a function of time for different indentations. The stick-slip phenomenon was found during the sliding motion and a 3 mm indentation generated the smallest stick-slip magnitude. We considered the largest deflection value from the stick-slip curve as the simulated bending deflection and compared the results at different contact indentations (see Figure 4D). We found that the bending deflection quickly converged to over 4 mm after an indentation of 1 mm.

### 3.3 Experimental Test

**FIGURE 5:**
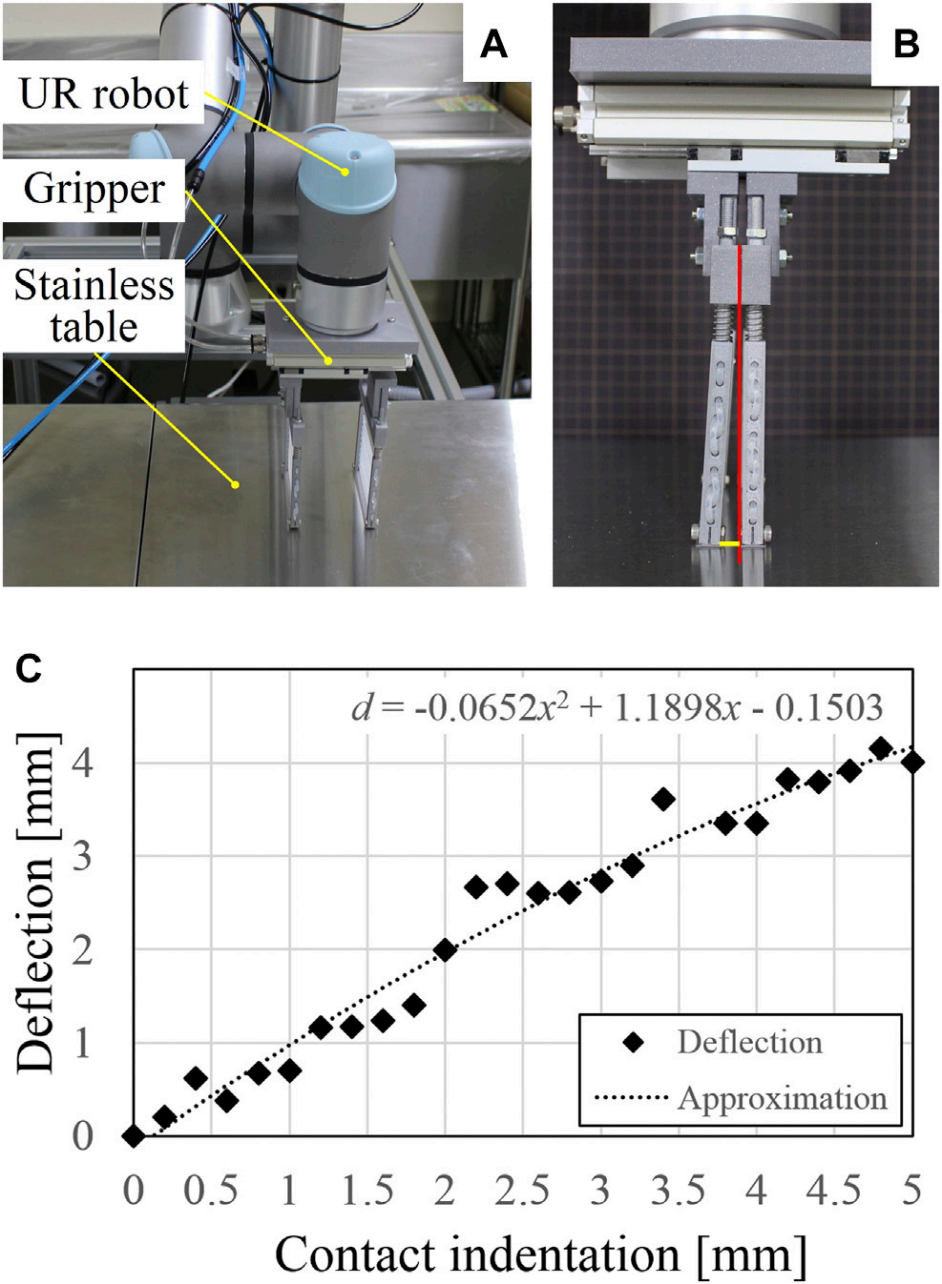
**(A)** Experimental setup for the contact tests. **(B)** Example of the bending deflection of the rigid rod at an indentation of 5 mm. **(C)** Relationship between the experimentally measured bending deflection and different contact indentations.

To validate the results of the contact analysis, we conducted contact tests using a robotic manipulator (UR5e, Universal Robots, Denmark). The experimental setup is shown in Figure 5A. The scooping-binding gripper was attached to the manipulator, and the position was adjusted to align with the stainless table. Then, the robotic manipulator was controlled to move downward at a predetermined distance to generate an indentation, after which the gripper was closed by applying compressed air into the pneumatic actuator and the rigid rods bent owing to the friction force. Figure 5B shows an example of the bending deflection of the rigid rod at an indentation of 5 mm. The rod on the right side was manually returned back to vertical for comparison. The bending deflection of the left rod was measured as the length of the thick yellow line in Figure 5B. The contact tests were conducted up to a 5 mm indentation with an indentation interval of 0.2 mm.

The relationship between the measured bending deflection and the contact indentation is shown in Figure 5C. The plot suggests an approximately second-order polynomial relationship as below.

$$d=-0.0789x^{2}+1.2365x-0.1735,$$ (2)

where deflection *d* and indentation *x* have the same definitions in Section 3. In experiment, the largest bending deflection, *i.e.,* 4.15 mm, was reached at an indentation of 4.8 mm. This value of largest bending deflection is similar to that provided by finite element simulation, but the convergence of the deflection is not evident in the experimental results. Compared with the calculated results using the simple beam model (see Figure 3), the increasing tendencies are similar, but the experimental measurements showed much less deflections. This may be due to the contact condition between the thin plate and the stainless table. In the experiments, the thin plate may not be perfectly aligned with the table, and the friction coefficient may change depending on the contact condition. Nevertheless, the results of the contact tests suggest that an indentation below 5 mm does not significantly affect the closing performance of the gripper. Therefore, the gripper is able to accommodate position errors in the vertical direction while contacting with the external environment.

## 4 Binding Force Analysis

### 4.1 String Characterization

**FIGURE 6:**
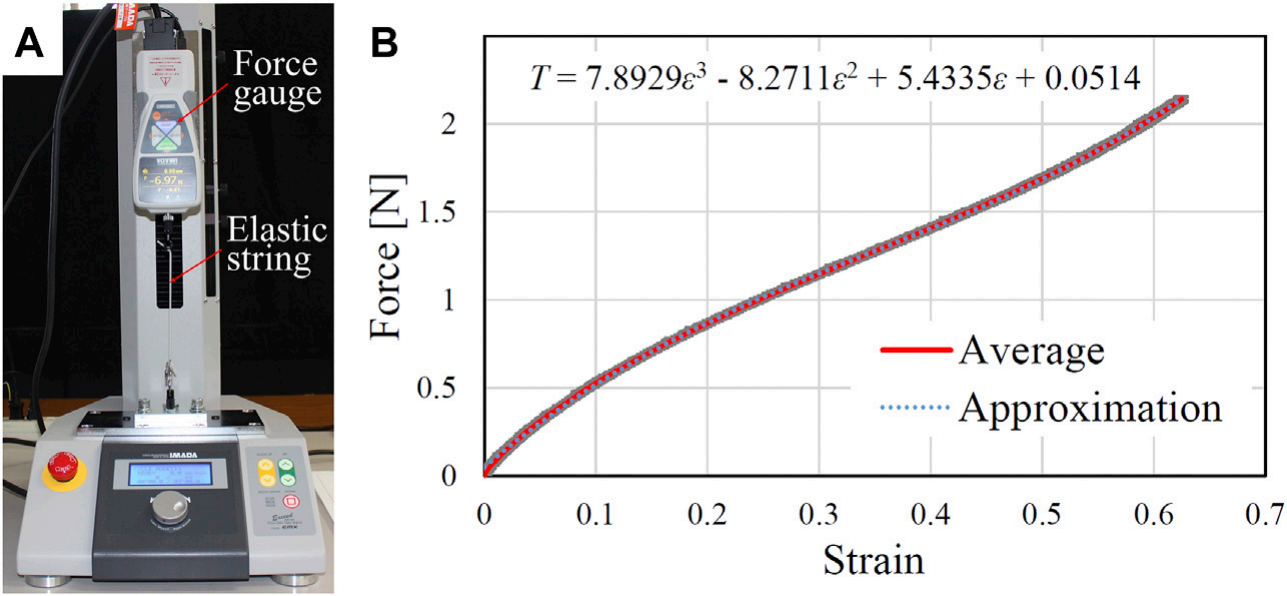
**(A)** Experimental setup for the flexible string characterization. **(B)** Relationship between the strain and the tension force. The shaded region indicates the standard deviation from five trials.

To characterize the tension of the flexible string, we conducted tensile test using a force-displacement measurement unit (FSA-1KE-50N, IMADA, Aichi, Japan). The experimental setup is shown in Figure 6A. The string was fixed between the end of the force gauge and the test stand. The force gauge was slowly translated upward with a velocity of 60 mm/s to extend the string. The tension force was recorded by the force gauge. Five trials were conducted and the relationship between the force and the strain is shown in Figure 6B. The standard deviation among five trials is small and we fitted the averaged force-strain curve using a third order polynomial equation as below.

$$T=7.8929\varepsilon^{3}-8.2711\varepsilon^{2}+5.4335\varepsilon +0.0514,$$ (3)

where *T* and *ε* denote the tension force and strain, respectively.

### 4.2 Binding Force Estimation

**FIGURE 7:**
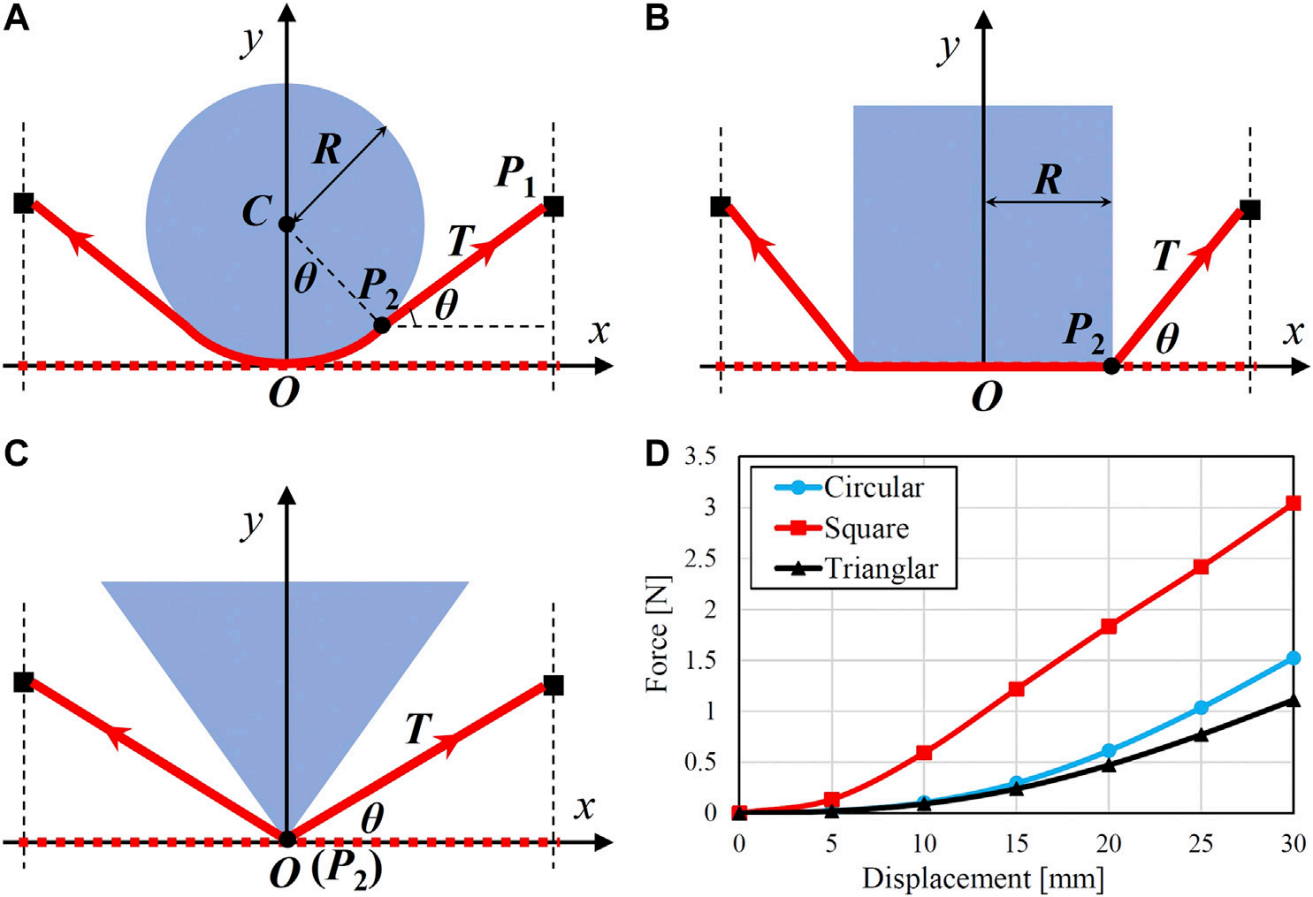
Model diagram for calculating the binding force while grasping a **(A)** circular, **(B)** square, and **(C)** triangular object, respectively. **(D)** depicts the calculated binding forces against different binding displacements.

To determine appropriate binding displacement for a known object, we established analytical models to estimate binding force generated by the flexible string during grasping. Three different shaped objects were considered (see Figures 7A–C). The undeformed and deformed string is indicated by the red dashed and solid line, respectively. Upon closing of the gripper, the right rod translated to point $P_{1}\left(L,d\right)$, where *L* and *d* indicate the half length of the undeformed string and the binding displacement, respectively. Point $P_{2}\left(x,y\right)$ denotes the last contact point between the object and the string. In the case of a circular object (see Figure 7A), the coordinates (x,y) can be determined by the following two equations:

$$\begin{array}{l} \left(L-x\right)x+\left(d-y\right)\left(y-R\right)=0,\\ x^{2}+{\left(y-R\right)}^{2}-R^{2}=0. \end{array}$$ (4)

The first equation defines the perpendicular relationship between the two vectors of $\vec{P_{1}P_{2}}$ and $\vec{CP_{2}}$. The second equation describes the distance between the point $P_{2}$ and the center of the object. For the square and triangular objects, the coordinates of contact point can be easily obtained as (R,0) and (0,0) according to Figures 7B,C, respectively. By knowing the coordinates of $P_{2}$, we can calculate the angle θ as

$$\begin{array}{l} \mathrm{Circular}:\theta_{c}=\arctan\frac{d-y}{L-x},\\ \mathrm{Square}:\theta_{s}=\arctan\frac{d}{L-x},\\ \mathrm{Triangular}:\theta_{t}=\arctan\frac{d}{L}. \end{array}$$ (5)

The deformed length ($L_{c}$, $L_{s}$, $L_{t}$) and strain ($\varepsilon_{c}$, $\varepsilon_{s}$, $\varepsilon_{t}$) of half string can be then calculated by

$$\begin{array}{l} \mathrm{Circular}:L_{c}=R\theta_{c}+\frac{d-y}{\sin\theta_{c}},\;\varepsilon_{c}=\frac{L_{c}-L}{L}\\ \mathrm{Square}:L_{s}=R+\frac{d}{\sin\theta_{s}},\;\varepsilon_{s}=\frac{L_{s}-L}{L}\\ \mathrm{Triangular}:L_{t}=\frac{d}{\sin\theta_{t}},\;\varepsilon_{t}=\frac{L_{t}-L}{L}. \end{array}$$ (6)

where *R* denotes the radius of the circular object and the half length of the square object. The tension force of the deformed half string can be then obtained by substituting the strain values into Eq. 3. If we suppose the contact between the object and the string as non-slip condition, we can ignore the effect of friction. Therefore, we can finally calculate the binding force generated by the extension of the string as

F=2T⁡sin⁡θ (7)

Based on the above analysis, we conducted simulations by supposing the parameters of *L* = 42 mm, *d* varying from 5 to 30 mm with an interval of 5 mm, and *R* = 30 mm. The calculated binding forces are shown in Figure 7D. At the same binding displacement *d*, grasping a square object generates much larger binding force comparing to a circular or triangular object.

### 4.3 Binding Force Test

**FIGURE 8:**
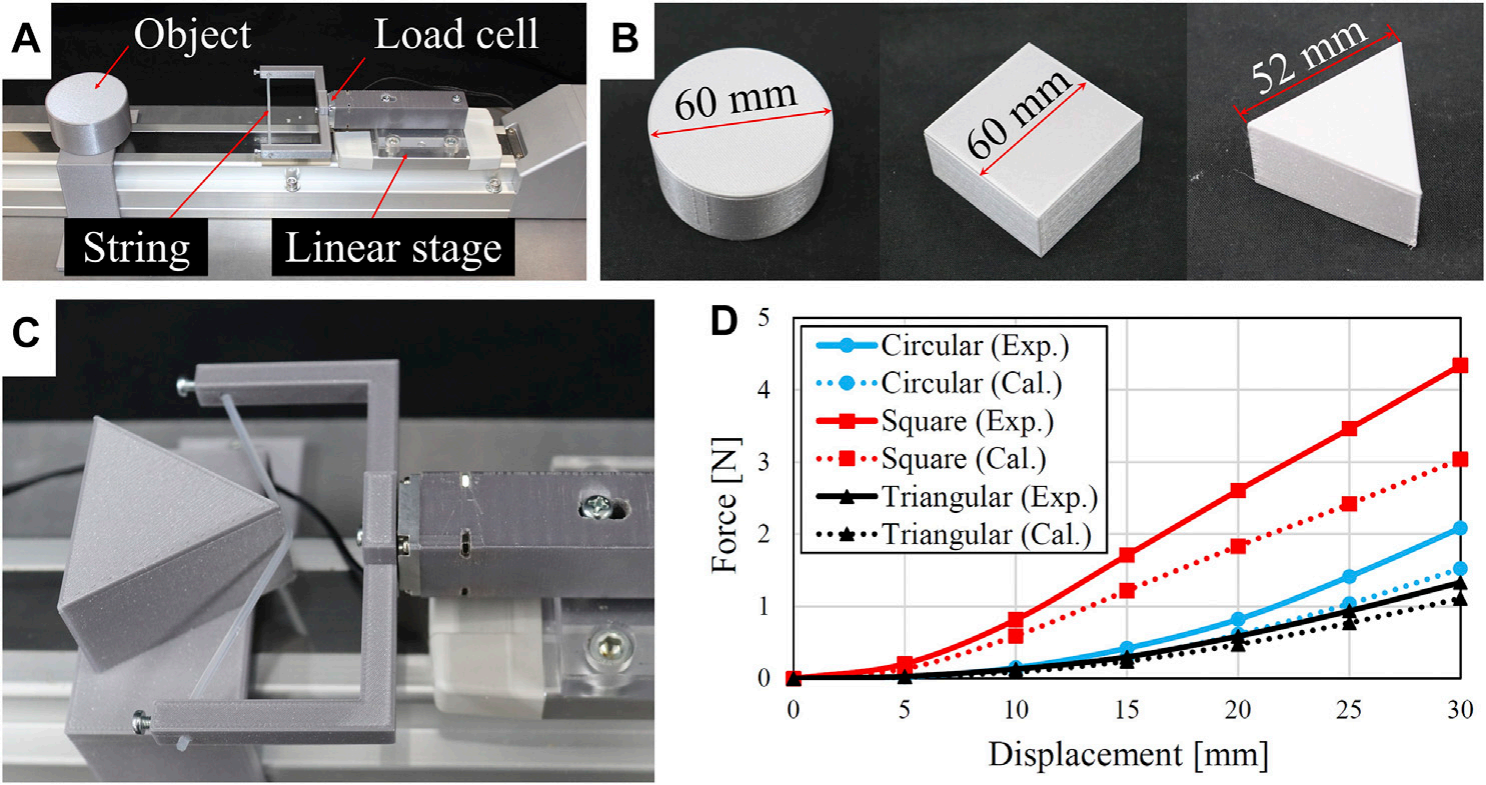
Bending force tests: **(A)** experimental setup, **(B)** three objects for the tests, **(C)** test scenario on the triangular shaped object, and **(D)** comparisons of binding force between experiments and calculations.

To validate the analytical model of the binding force, we performed the binding force tests using the experimental setup shown in Figure 8A. The flexible string was fixed on a 3D printed U-shaped frame, which was further fixed on a load cell (USL06-H5-50N, Tec Gihan, Kyoto) for measuring the applied force. A linear actuator (LEFS32B-500, SMC, Tokyo) was used to translate the string towards the object to generate a binding deformation. We tested three objects with circular, square, and triangular shapes (see Figure 8B). Each object was tested five times and the binding displacement *d* was tested up to 30 mm. One example of the binding test is shown in Figure 8C. We compared the measured binding forces to the calculated ones (see Figure 8D). The tendencies are very similar but the calculated binding forces have smaller amplitudes than that of the measured ones, and the discrepancy is less at lower binding displacements. The discrepancy may be caused by the friction between the object and the string and friction effect is more obvious at larger displacements beacuse of the larger tension forces. Nevertheless, we can use the established analytical model to roughly predict the binding force based on the object geometry and the binding displacement. Therefore, it is possible to determine an appropriate binding displacement of the gripper to not damage an fragile food product.

## 5 Food Handling Experiments

**FIGURE 9:**
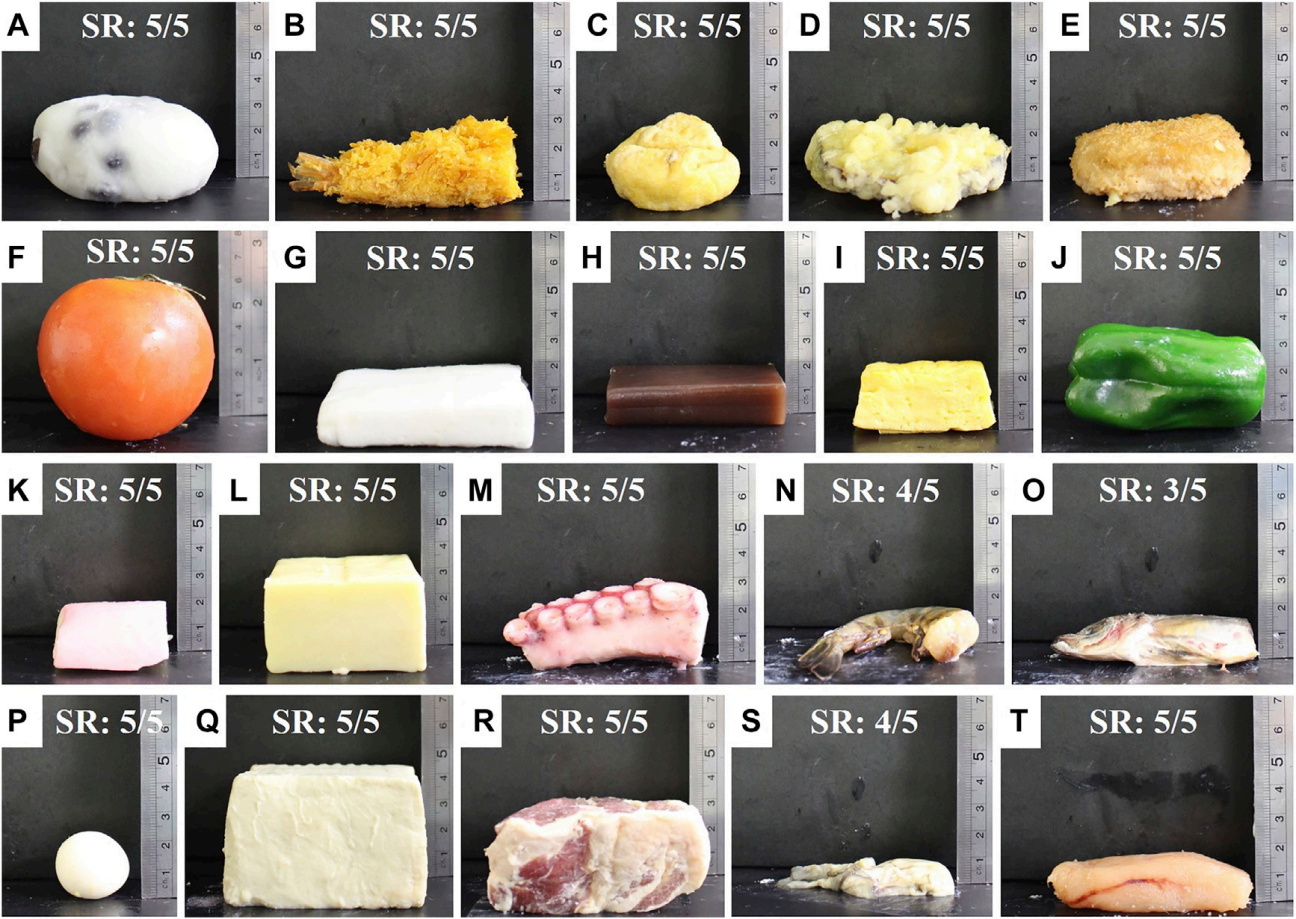
Food items used in the grasping experiments: daifuku (small round mochi stuffed with sweet red bean paste) **(A)**, fried shrimp **(B)**, ganmodoki (deep-fried tofu dumpling) **(C)**, sweet potato tempura **(D)**, croquette **(E)**, tomato **(F)**, boiled flat fish cake **(G)**, sweet bean jelly **(H)**, rolled omelet **(I)**, green pepper **(J)**, kamaboko (steamed fish cake) **(K)**, cheese **(L)**, octopus leg **(M)**, raw shrimp **(N)**, raw carangidae fish **(O)**, quail egg **(P)**, tofu **(Q)**, meat **(R)**, raw oyster **(S)**, and Pollock roe **(T)**. “SR” indicates the success rate of the handling tests.

To evaluate the capability of the scooping-binding gripper, handling tests on various food items were conducted using a 4-DOF SCARA robot (HSR065A1-N, DENSO WAVE, Aichi). A total of 20 food items (see Figure 9) were used in the experiments. The approximate dimensions, weights, and physical property descriptions of these food items are listed in Table 2. The food items included fried foods (**B**, **C**, **D**, **E**), very soft foods (**A**, **I**, **Q**, **S**, **T**), relatively heavy foods (**A**, **F**, **Q**), fragile foods (**S**, **T**), foods with thin profiles (**D**, **E**, **G**, **H**, **N**, **O**, **S**, **T**), and food with slippery surfaces (**G**, **M**, **N**, **O**, **S**, **T**). Some of them, such as the raw oyster (**S**) and the Pollock roe (**T**), combine several properties that are difficult for robotic grippers to manage.

**TABLE 2:** Physical properties of the food items used in the handling tests. The size is described as Length x Width x Height; the maximum value of each dimension is provided. Softness and slipperiness were qualitatively rated by star marks with five stars indicating the strongest properties. Bold letters of A, B, C, ... correspond to the food item labels in Figure 9.

Food	Size [mm]	Weight [g]	Softness	Slipperiness
Daifuku **(A)**	69 x 67 x 30	99.1	***	***
Fried shrimp **(B)**	70 x 25 x 25	16.9	**	***
Ganmodoki **(C)**	52 x 37 x 17	19.6	***	***
Potato tempura **(D)**	64 x 48 x 10	28.1	**	***
Croquette **(E)**	65 x 47 x 20	40.7	***	***
Tomato **(F)**	73 x 70 x 65	188.7	*	**
Boiled fish cake **(G)**	80 x 55 x 18	36.5	***	****
Sweet bean jelly **(H)**	50 x 37 x 16	35.2	***	**
Rolled omelet **(I)**	47 x 29 x 23	25.7	****	****
Green pepper **(J)**	70 x 43 x 42	28.2	*	**
Steamed fish cake **(K)**	49 x 47 x 19	33.9	***	****
Cheese **(L)**	55 x 50 x 31	99.0	***	**
Octopus leg **(M)**	88 x 39 x 23	55.7	*	****
Raw shrimp **(N)**	69 x 50 x 16	14.5	*	*****
Carangidae fish **(O)**	52 x 30 x 17	15.5	*	*****
Quail egg **(P)**	29 x 26 x 25	9.5	***	****
Tofu **(Q)**	54 x 50 x 34	150.8	****	*****
Meat **(R)**	47 x 44 x 31	55.3	***	****
Raw oyster **(S)**	55 x 26 x 15	10.2	*****	*****
Pollock roe **(T)**	55 x 26 x 18	23.5	*****	****

**FIGURE 10:**
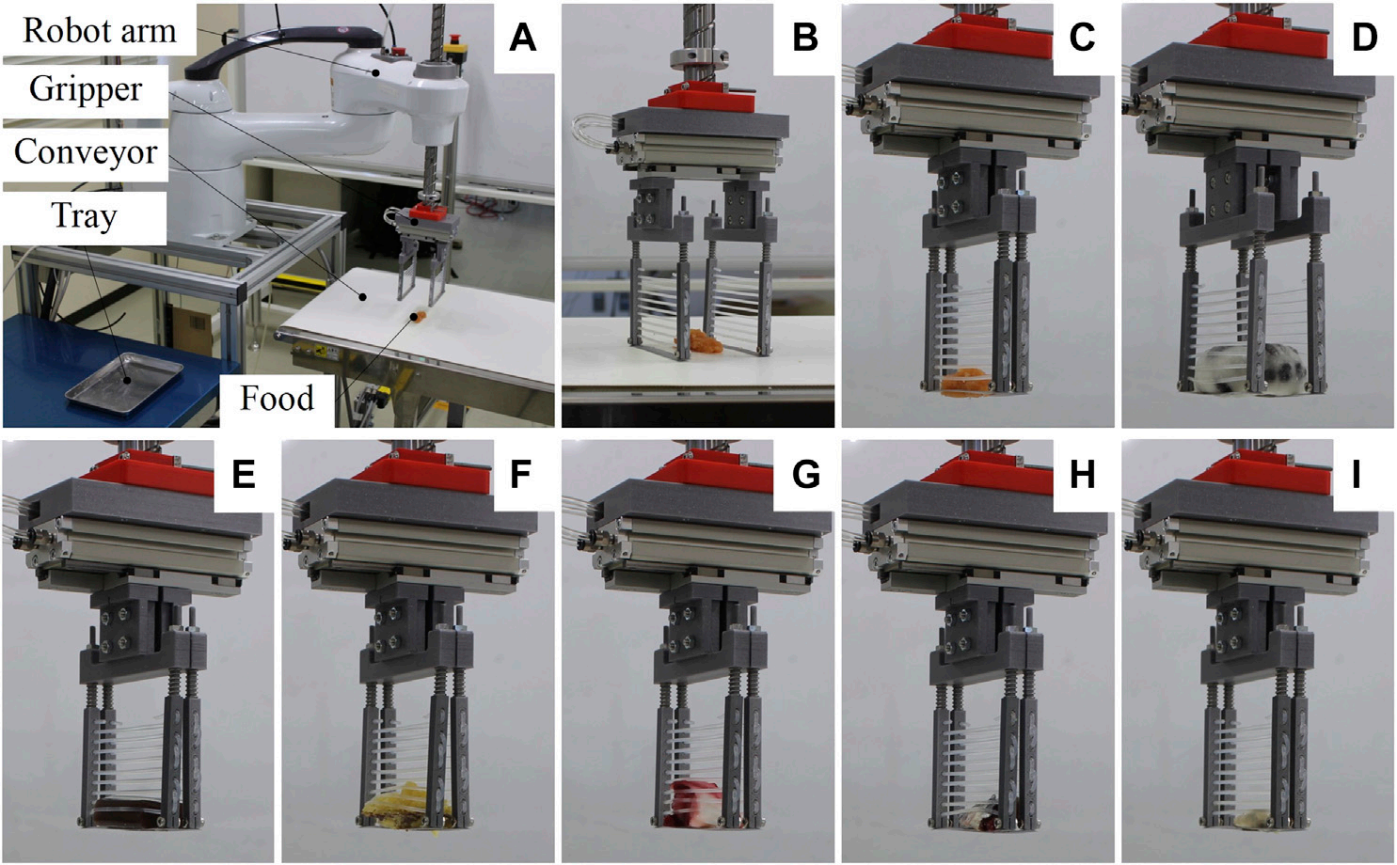
**(A)** Experimental setup of the handling test **(B)** Opening state before grasping. Experimental snapshots while grasping and lifting the Pollock roe **(C)**, daifuku **(D)**, sweet bean jelly **(E)**, sweet potato tempura **(F)**, octopus leg **(G)**, carangidae fish **(H)**, and raw oyster **(I)**, respectively.

The handling test is a conventional pick-and-place operation frequently adoped in the food industry for packaging. The experimental system is shown in Figure 10A. The SCARA robot was used because of its large payload and ability to perform high-speed motion. The scooping-binding gripper was attached to the SCARA robot using a 3D printed flange interface (the component in red color). A conveyor suitable for food conveying (FB2C-UD-309-400-300-IV-25, Maruyasu Kikai, Nagano) was used to place the food items. A pick-and-place motion was programmed, and the gripper picked the food item and placed it into the stainless tray. The maximum operation speed of the robot was used to test the stability of the grasping. An indentation of 1 mm was used for the tests. A pneumatic system including an air compressor (SLP-15EFDM6, Anest Iwata, Yokohama), pressure regulator (ITV2030, SMC, Tokyo), and solenoid valves (VQ110U-5M-M5, SMC, Tokyo) was used to actuate the gripper. Five trials were conducted for each food item, and the posture of the food for each trial was manually adjusted to account for an arbitrary grasping posture.

Some experimental snapshots while grasping various food items are shown in Figures 10B–I, and the handling success rate (number of success trial/total trial number) of all food items are listed in Table 3. Supplementary Video S1 demonstrates the performances of the gripper handling all food items. The gripper succeeded in handling all food items with a success rate of 100% except for the raw shrimp (80%), the carangidae fish (60%), and the raw oyster (80%). The results demonstrate that the scooping-binding gripper could handle all food items despite their complex geometries, thin profiles, slippery surface conditions, and variations in shape, size, and softness. For handling daifuku, tomato, tofu, and meat, a wide configuration of the gripper was used because the size of the products.

**TABLE 3:** Success rate (number of succeeded trials/total trial number) of the handling tests, where “Wide” and “Narrow” denote different gripper configurations. Bold letters of A, B, C, ... correspond to the food item labels in Figure 9.

Food	Configuration	Rate	Food	Configuration	Rate
Daifuku **(A)**	Wide	5/5	Fried shrimp **(B)**	Narrow	5/5
Ganmodoki **(C)**	Narrow	5/5	Potato tempura **(D)**	Narrow	5/5
Croquette **(E)**	Narrow	5/5	Tomato **(F)**	Wide	5/5
Boiled fish cake **(G)**	Narrow	5/5	Sweet bean jelly **(H)**	Narrow	5/5
Rolled omelet **(I)**	Narrow	5/5	Green pepper **(J)**	Narrow	5/5
Steamed fish cake **(K)**	Narrow	5/5	Cheese **(L)**	Narrow	5/5
Octopus leg **(M)**	Narrow	5/5	Raw shrimp **(N)**	Narrow	4/5
Carangidae fish **(O)**	Narrow	3/5	Quail egg **(P)**	Narrow	5/5
Tofu **(Q)**	Wide	5/5	Meat **(R)**	Wide	5/5
Raw oyster **(S)**	Narrow	4/5	Pollock roe **(T)**	Narrow	5/5

The gripper failed one trial for handling raw shrimp and raw oyster, respectively, and two trials for handling the carangidae fish. This was due to the slippery property and minimum height profiles of these food items. Their maximum heights are higher than the gap between the lowest rubber string and the thin plate, but their minimum heights are smaller than that gap. At a certain grasping orientation, the food item may slip out through the gap due to the slippery surface. This problem can be solved by reducing the gap between the lowest string and the thin plate and increasing the friction coefficient of the string and the thin plate.

The takt time of the handling tests was approximately 4 s, and the results validated the stability of the scooping-binding gripper working at high-speed motion. During the experiments, significant damage to the food items was not found because of the flexibility of the rubber string. The takt time depends on the travel distance of the pick-and-place motion and it can be further reduced by using a faster robot manipulator, such as a parallel robot. Multi-gripper system being able to handling multiple food products at once is another solution to shorten the takt time.

## 6 Conclusion

In this study, we propose a scooping-binding gripper for handling various food products including those with thin profiles and slippery surfaces. The gripper actively makes contact with the external environment for grasping. The gripper employs thin plates to scoop the food product from its bottom and utilizes thin rubber strings to bind the food product to stabilize the grasp. The contact behavior of the scooping-binding mechanism was analyzed using the simple beam theory and finite element method, and was experimentally validated by contact tests. The results show that the finite element model can predict the largest bending deflection of the scoop-binding mechanism upon gripper closing. The mechanism bent less than 5 mm at an indentation of 5 mm. This suggests that the gripper can accommodate at least a 5 mm position error in the contact direction.

The tension property of the flexible string was characterized and an analytical model was proposed to estimate the binding force of the flexible string based on the object geometry and the binding displacement. Therefore, in order to not damage the food product while grasping, it is possible to predetermine an appropriate binding displacement according to the approximated geometry and phsyical property of the food product.

Experimental tests on handling 20 food items were conducted to demonstrate the capability of the proposed gripper. This set of food items includes products with thin profiles, such as potato tempura (height of 10 mm), raw oyster (height of 15 mm), and raw shrimp (height of 16 mm); products with slippery surfaces, such as boiled fish cake, carangidae fish, and raw oyster, and products with fragile properties, such as tofu, raw oyster, and Pollock roe. We found that the proposed gripper could handle all the food items despite their complex physical properties owing to the combination of the scooping and binding abilities. By contrast, the gripper failed few trials for handling raw shrimp, carangidae fish, and raw oyster because of the relatively large gap between the thin plate and the lowest string. This can be improved by revising the design of the rigid rod to reduce the gap.

This paper presents a proof-of-concept of the scooping-binding gripper for handling various food products despite of their thin profiles and complex physical properties. In the future, camera will be used to recognize a food item and extract its geometry, and stroke-controllable actuator will be used to drive the scooping-binding mechanisms to adjust the binding displacement accoding to the recognized food geometry.

One limitation of the current gripper is food compatibility. According to the EHEDG (European Hygienic Engineering and Design Group) guidelines (EHEDG, 2018), stainless steels are the logical preference to construct equipment for product contact, and fasteners which may loosen and fall into product should be elimnated. Therefore, stainless steel will be used to manufacture the rigid parts of the gripper and welding will be used to connect the rigid rod and thin plate. For the flexible string, food-compatible elastomers will be investigated and selected in the future.

## Data Availability

The original contributions presented in the study are included in the article/Supplementary Material, further inquiries can be directed to the corresponding author.
